# Prognostic significance of inflammatory indices in hepatocellular carcinoma treated with transarterial chemoembolization: A systematic review and meta-analysis

**DOI:** 10.1371/journal.pone.0230879

**Published:** 2020-03-26

**Authors:** Shuangshuang Li, Xudong Feng, Guodong Cao, Qianhui Wang, Ling Wang

**Affiliations:** 1 Department of Microbiology and Center of Infectious Disease, School of Basic Medical Sciences, Peking University Health Science Center, Beijing, People’s Republic of China; 2 State Key Laboratory for the Diagnosis and Treatment of Infectious Diseases, The First Affiliated Hospital, College of Medicine, Zhejiang University, Hangzhou, Zhejiang province, People’s Republic of China; 3 Department of Surgery, Second Affiliated Hospital, School of Medicine, Zhejiang University, Hangzhou, Zhejiang Province, People’s Republic of China; 4 Department of Infectious Diseases, Taiyuan No. 3 Hospital, Taiyuan, Shanxi Province, People’s Republic of China; The University of Tokyo, JAPAN

## Abstract

**Objectives:**

To investigate the association between inflammatory indices and clinical outcomes of hepatocellular carcinoma (HCC) patients treated with transarterial chemoembolization (TACE) by performing meta-analysis.

**Methods:**

A systematic literature search for relevant studies published up to August 2019 was performed by using PubMed, Web of Science, EMBASE, China National Knowledge Internet (CNKI) and Wanfang databases. Pooled hazard ratios (HR) or odds ratio (OR) and 95% confidence intervals (95% CI) were calculated.

**Results:**

A total of 5280 patients from 22 studies were finally enrolled in the meta-analysis. The results demonstrated that elevated preoperative NLR, PLR, and CRP was associated with poor OS in HCC patients treated by TACE (HR = 1.81, P<0.00001; HR = 1.56, P = 0.007; HR = 1.45, P<0.00001, respectively). In addition, high NLR was significantly correlated with the presence of tumor vascular invasion (OR = 1.49, P = 0.002). Elevated PLR tended to be correlated with higher incidence of tumor size>3 cm (OR = 2.42, P = 0.005).

**Conclusions:**

Elevated preoperative NLR, PLR, and CRP are associated with poor prognosis in HCC patients treated with TACE. These inflammatory indices may be convenient, accessible, affordable and dependable biomarkers with prognostic potential for HCC patients treated by TACE.

## Introduction

Hepatocellular carcinoma (HCC), a highly aggressive and prevalent tumor with increasing incidence rate over the last several decades, is the seventh most common malignant tumors worldwide and the fourth leading cause of cancer-related mortality[1]. Resection, liver transplantation may be curative for the early stage of tumor, which accounts for ≤ 30% of patients[2]. However, most of patients with hepatocellular carcinoma are initially diagnosed at an intermediate to advanced stage, where hepatic resection and liver transplantation are not feasible[3]. Transarterial chemoembolization (TACE) is considered to be the standard treatment for patients at intermediate stage according to the Barcelona Clinic Liver Cancer classification (BCLC) stage[4]. TACE can be used to treat well-compensated cirrhosis patients, which can reduce their burden of disease and potentially prolong their life. It is a non-surgical, minimally invasive and well-tolerated procedure with acceptable morbidity[5]. A few criteria have been proposed to predict the prognosis of patients and to help clinicians design optimal personalized treatment strategies, like Tumor Node Metastasis (TNM), functional liver reserve, Cancer of the Liver Italian Program (CLIP) staging score and Barcelona Clinic Liver Cancer (BCLC) score[6]. However, due to the tedious content of these standards, there are many inconveniences in practical applications. Although these criteria are mostly efficient in predict patients prognosis, they add a lot of burden to clinicians and patients, which explains why they are rarely used in routine clinical practice[6]. Therefore, it is essential to identify effective, common and easy-obtained prognostic biomarkers, especially simple serum biomarkers for prognosis of HCC undergoing TACE.

Homogeneous inflammation is vital for health; insufficient inflammation may lead to persistent infection of pathogens, while excessive inflammation may cause chronic or systemic inflammatory diseases[7]. Since the discovery of the close relationship between inflammation and malignancy in 1863, increasing evidence has suggested that the presence of a systemic inflammatory response is highly correlated with poor prognosis for malignancies[8–10]. Moreover, the presence of a systemic inflammatory response can be detected by C-reactive protein (CRP) and inflammation-related cells, including neutrophils, lymphocytes, and platelets. NLR values represent the absolute neutrophil count divided by the absolute lymphocyte count. PLR values represent the absolute platelet count divided by the absolute lymphocyte count. Thus, a variety of inflammatory indices such as CRP, neutrophil to lymphocyte ratio (NLR), platelet to lymphocyte ratio (PLR), modified Glasgow prognostic score (mGPS) and prognostic nutritional index (PNI) have been proposed and have been proven to have prognostic value in multiple cancers[11–17]. However, as a matter of contradictory results as well as the small sample size in solitary study, the current opinion of inflammatory indices as the prognostic markers in HCC patients treated with TACE is still inconclusive.

We therefore collected the eligible studies and conducted this meta-analysis to investigate the relationship between some novel inflammatory indices and the prognosis of HCC patients treated with TACE.

## Materials and methods

### Literature search strategy

The following databases were systematically searched until August 2019 without time restrictions: PubMed, Web of Science, EMBASE, China National Knowledge Internet (CNKI) and Wanfang databases. The search strategy was based on combination of following terms:(“NLR” or “neutrophil to lymphocyte ratio” or “neutrophil-lymphocyte ratio” or “PLR” or “platelet to lymphocyte ratio” or “platelet-lymphocyte ratio” or “C-reactive protein” or “CRP” or “prognostic nutritional index” or “PNI”) AND (“hepatocellular carcinoma” or “HCC” or “liver carcinoma” or “liver cancer”) AND (“TACE” or “transarterial chemoembolization”). References cited in the retrieved articles were also scanned for relevant studies. Two assessors independently screened the title and abstract of each study. Once relevant studies became certain, the full texts were obtained for further evaluation.

### Selection and exclusion criteria

Studies included in the meta-analysis had to meet the following criteria: (1) HCC was diagnosed by pathological methods; (2) inflammatory indices was measured by serum-based methods before TACE treatment; (3) hazard ratios (HR) and 95% confidence intervals (95% CI) for different inflammatory indices in overall survival (OS) were described in the study or could be calculated from the supplied data. The exclusion criteria were as follows: (1) letters, reviews, comments, conference abstract, full text not available; (2) articles without deficit cutoff value of indices; (3) overlapping or duplicate data; (4) TACE combined with sorafenib treatment.

### Data extraction and quality assessment

Data were extracted separately by two reviewers, and disagreement was resolved by joint discussion. The following data of each study were recorded: name of first author, year of publication, research time, country, sample size, patients’ age, gender, BCLC stage, Child-Pugh score, treatment methods, inflammation indices, cut-off value, time of follow-up, survival data, and clinicopathologic parameters. The quality of the included studies was assessed using the 9-star Newcastle-Ottawa Scale (NOS) by two independent reviewers. The NOS consists of three aspects: selection, comparability, and outcome assessment between the case group and the control group. Studies with the NOS scores ≥ 6 were regarded as high-quality studies. The consensus about the quality of studies was achieved as disagreement between the two reviewers was resolved through discussion.

### Data analysis

HR and their associated standard errors (SE) were pooled to give the effective value for the quantitative aggregation of the survival results. When these statistical variables are not directly available in the original article, they were calculated from the available data using methods reported by Parmar et al[18]. For the pooled analysis of the relationship between inflammation index (NLR, PLR) and clinical features, odds ratio (OR) and their 95% CI were pooled to give the effective value.

The Review Manager version 5.3 and STATA software (version 12.0) were used for data analysis. The heterogeneity between studies was assessed by the chi-squared and I-squared tests. If heterogeneity was significant (I^2^ ≥ 50%), random-effect model was used to calculate the pooled HR and 95% CI. Otherwise, fixed-effect model was performed. All P values were two-tailed with a significant level at 0.05. Log (HR) and their associated standard errors (selog(HR)) were pooled to conduct the Begg's funnel plots. Begg's rank correlation was used to determine potential publication bias. P value less than 0.05 indicates statistically significant publication bias.

## Results

### Literature information

**Fig 1:**
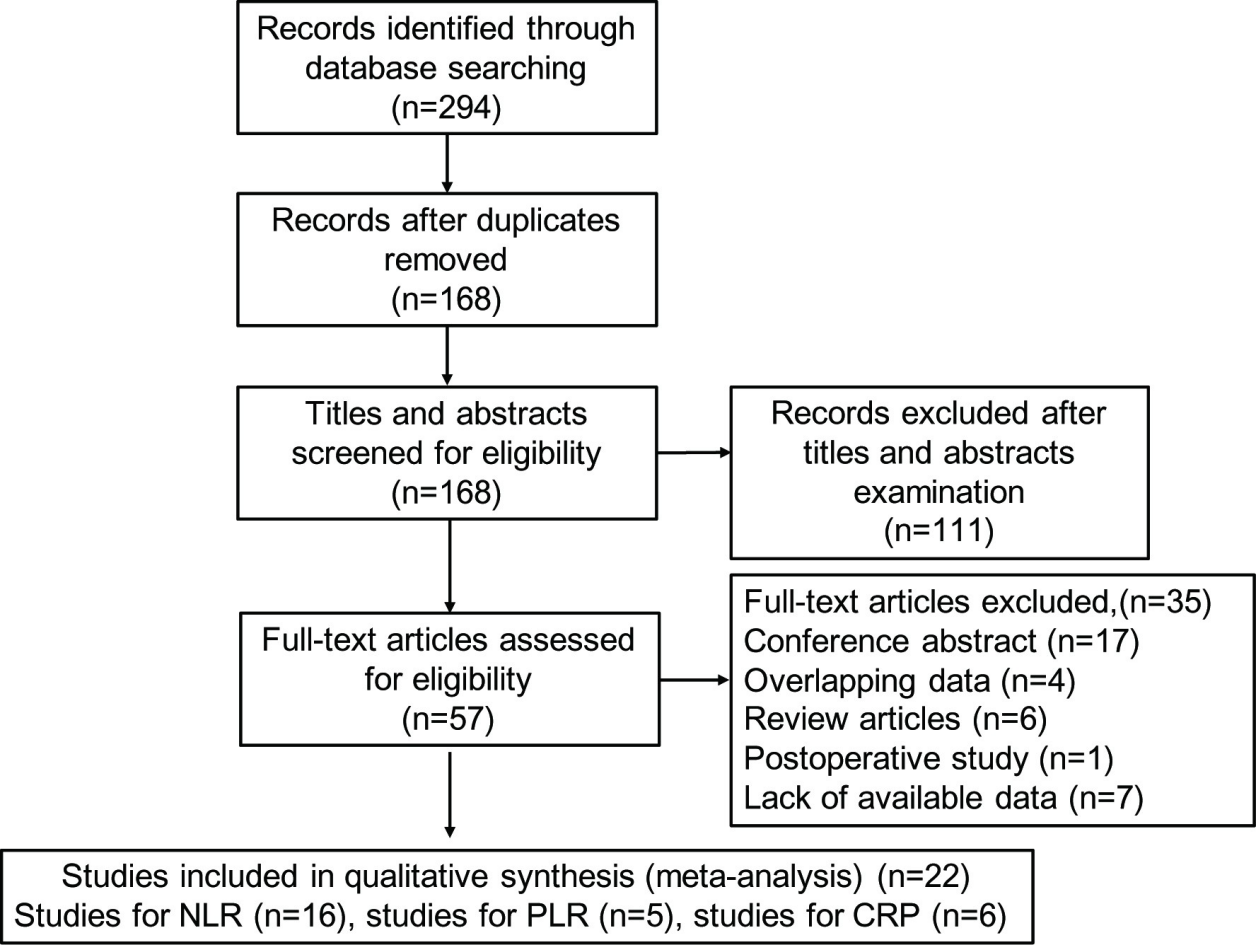
Flow chart of the study selection procedure.

In total, 294 potentially relevant records were initially identified after searching PubMed, Web of Science, EMBASE, CNKI and Wanfang databases (Fig 1). After removal of duplicates, 168 studies were selected and screened for eligibility. Of these, 111 irrelevant records were excluded after screening the titles or abstracts. After carefully reading the full text of the remaining 57 studies, 35 papers that did not meet the inclusion criteria were further excluded. Subsequently, 22 studies were included in qualitative synthesis.

### Characteristics of included studies

A total of 22 studies[2,5,19–38] published between 2011 and 2019 were identified and all these trials were retrospective cohort studies with 5280 patients enrolled in this meta-analysis. The basic characteristics of the included studies were summarized and presented in Table 1. Sixteen studies were conducted in China, the other six studies were conducted in Korea, Australia, German, USA, Japan, Italy, respectively. All the studies were HCC patients received TACE and OS data were reported or estimated. Sample sizes ranged from 49 to 921. Of the 22 studies, 16 of studies[2,5,19,21,23–25,27–34,37] were about the prognostic value of preoperative NLR for OS, 5 about PLR[23,24,26,32,33] and 6 about CRP [20,22,33,35,36,38]. The NLR cut-off values in these studies were determined by different methods and ranged from 1.77 to 5. The cut-off values used for PLR ranged from 94.62 to 150. The cut-off values used for CRP ranged from 0.5 to 1 mg/dl.

**Table 1:** Characteristics of included studies in this meta-analysis.

Author/ year	Country	Treatment	Sample size (n, male)	BCLC stage	Child-Pugh class	Sampling time	Mean/median ages (years)	Follow-up time (months)	Inflammation index	Cut-off value	Outcome	NOS score
Chon 2019	Korea	cTACE	921(700)	A/B/C	A/B	before TACE	68.2	13–61.4	NLR	NLR = 5	OS	7
Fan 2015	China	cTACE	132(87)	NA	A/B	before TACE	49	4–46	NLR/PLR	NLR = 3.1 PLR = 137	OS	7
He 2019	China	cTACE	216(200)	B/C	A/B	before TACE	53	1–56	NLR/PLR/CRP	NLR = 1.77 PLR = 94.62 CRP = 0.8mg/dl	OS	8
Huang 2011	China	cTACE	145(134)	NA	A	before TACE	49	1–41	NLR	NLR = 3.3	OS	8
Hucke 2014	Australia	cTACE/DEB-TACE	131(115)	A/B	A/B	before TACE	66	NA	CRP	CRP = 1mg/dl	OS	7
Le 2019	China	cTACE	303(274)	C	A/B	before TACE	53	NA	CRP	CRP = 0.5mg/dl	OS	7
Li 2013	China	cTACE	154(134)	NA	A	before TACE	50	1–41	NLR	NLR = 2.5	OS	7
Li 2016	China	cTACE	117(86)	B/C	NA	before TACE	51.74	3–36	CRP	CRP = 1mg/dl	OS	7
Liu 2017	China	cTACE	760(643)	B/C	A/B	before TACE	56.5	1–69	NLR	NLR = 2.2	OS	8
Mahringer-Kunz 2017	German	cTACE/DEB-TACE	228(192)	A/B	A/B	before TACE	66.8	NA	CRP	CRP = 1mg/dl	OS	7
McNally2013	USA	cTACE/DEB-TACE	104(77)	NA	A/B/C	before TACE	56	1–56	NLR	NLR = 5	OS	7
Ogasawara 2015	JAPAN	cTACE	187(139)	B	A/B	before TACE	70	NA	CRP	CRP = 1mg/dl	OS	7
Rebonato 2017	Italy	cTACE/DEB-TACE	49(39)	B/C	A/B	before TACE	75	1–53	NLR	NLR = 2.03	OS/PFS	8
Sun 2018	China	cTACE	95(84)	B	A/B	before TACE	54.1	8–50	NLR/PLR	NLR = 2.51	OS	7
Tian 2016	China	cTACE	122(107)	NA	A/B	before TACE	56	NA	NLR/PLR	NLR = 2.61 PLR = 96.13	OS	7
Xu 2014	China	cTACE	178(149)	B	A/B	before TACE	54.3	1–99	NLR	NLR = 1.85	OS	8
Xue 2015	China	cTACE	291(258)	B/C	A/B	before TACE	53	1–61	PLR	PLR = 150	OS	8
Yang 2015	China	cTACE	546(453)	NA	A/B	before TACE	52	4–78	NLR	NLR = 3	OS	8
Zhang 2014	China	cTACE	138(99)	NA	A/B	before TACE	56.8	NA	NLR	NLR = 5	OS	7
Zheng 2013	China	cTACE	77(67)	B/C	A/B	before TACE	56.7	2–48	NLR	NLR = 4	OS	8
Zhou 2016	China	cTACE	279(251)	NA	A/B	before TACE	50	1–52	NLR	NLR = 2.6	OS	8
Zou 2017	China	cTACE	107(94)	B/C	NA	before TACE	50	1–100	NLR	NLR = 2	OS/DFS	5

TACE, transarterial chemoembolization; NLR, neutrophil-lymphocyte ratio; PLR, platelet to lymphocyte ratio; cTACE, conventional TACE; DEB-TACE, drug-eluting beads TACE

CRP, C-reactive protein; NA, not available; OS, overall survival; PFS, progression-free survival; DFS, disease-free survival; NOS score, Newcastle–Ottawa Quality Assessment Scale.

### Quality assessment

As Table 1 shows, there are nine studies with a NOS score of 8, twelve studies with a NOS score of 7, one study with a NOS score of 5 according to the NOS criteria. The mean score of the included studies was 7 (ranging from 5 to 8). Approximately 95% studies possessed good quality according to our definition for high-quality studies.

### The prognostic value of preoperative NLR for OS

**Fig 2:**
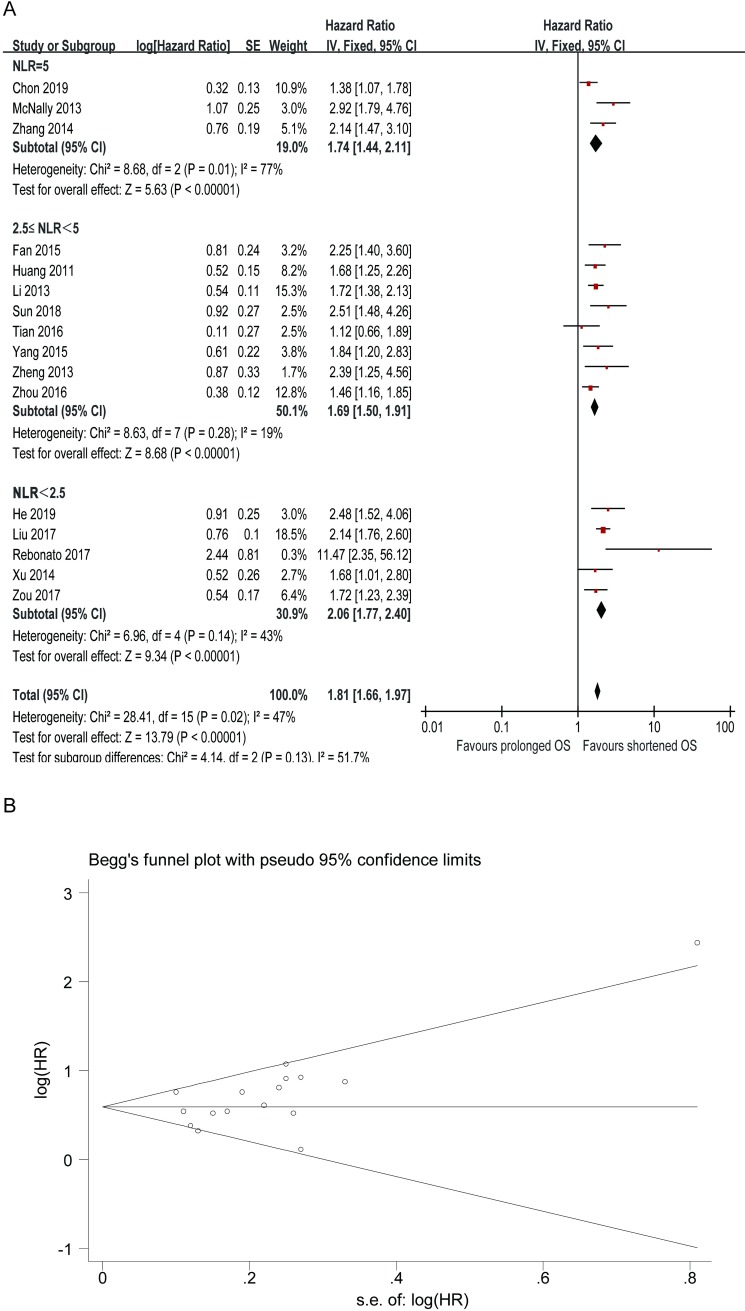
Correlation between NLR and overall survival of HCC treated by TACE. (A) Forest plot of comparison of the included trials. (B) Funnel plot of comparison of the included trials.

There were 16 studies investigating the association between preoperative NLR and OS of HCC patients who underwent TACE. Elevated preoperative NLR was significantly associated with poor OS with the pooled HR being 1.81 (95% CI: 1.66–1.97, P < 0.00001), demonstrating that elevated preoperative NLR was an indicator of poor survival rate in HCC patients initially treated with TACE (Fig 2A). Although heterogeneity was found among these studies (P = 0.02, I^2^ = 47%), the analysis was estimated using a fixed-effect model according to our model selection criteria. Subgroup analysis was also conducted to further investigate the prognostic effects of NLR on OS. In the subgroup, according to the cut-off value of NLR, statistically significance was found respectively in NLR = 5.0 (HR = 1.74, 95% CI: 1.44–2.11), 2.5 ≤ NLR <5 (HR = 1.69, 95% CI: 1.50–1.91), and NLR < 2.5 (HR = 2.06, 95% CI: 1.77–2.40). Surprisingly, in the NLR = 5.0 subgroup, the result indicated high statistical heterogeneity with an I^2^ value of 77% (P = 0.01), whereas no significant heterogeneity between studies was found in subgroup 2.5 ≤ NLR < 5 and subgroup NLR < 2.5, suggesting that NLR cut-off value for each study may be the source of heterogeneity of the pooled analysis. The funnel plot is used to assess the publication bias of the included literature, and we can roughly assess publication bias by observing whether its shape has any significant asymmetry. The funnel plot showed no clear evidence of publication bias, except that one trial was out of the symmetric region (Fig 2B). Begg’s test (P = 0.064) also provided a statistical evidence of the absence of significant publication bias.

### The prognostic value of preoperative PLR for OS

**Fig 3:**
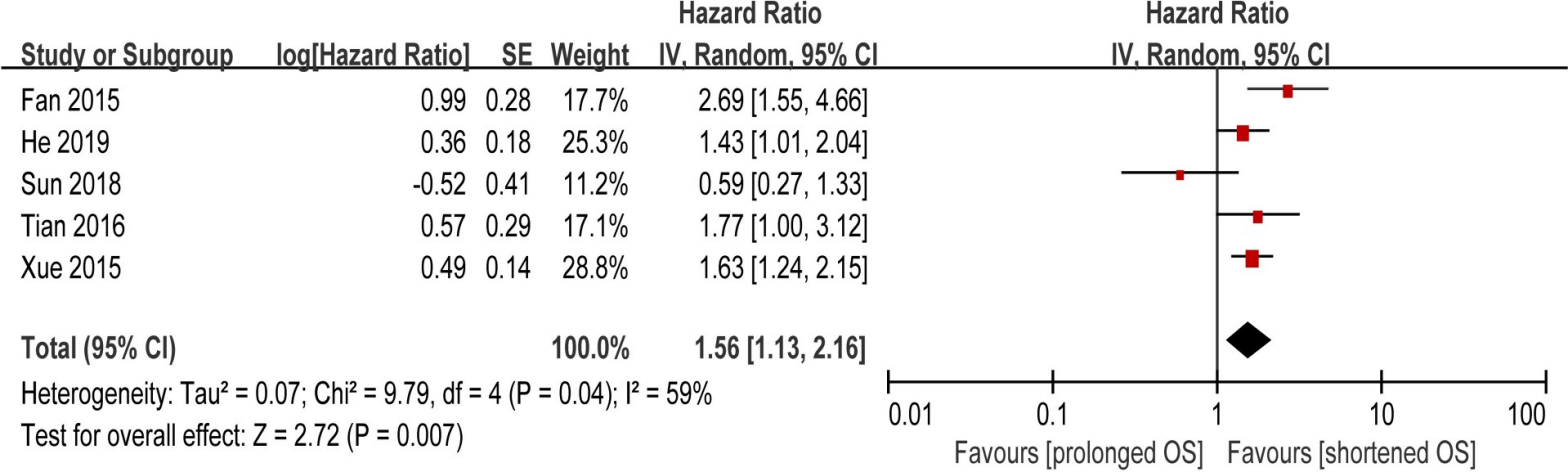
Forest plot od hazard ratio (HR) for the association of PLR with OS in HCC patients treated with TACE.

Five studies were included to evaluate the association between preoperative PLR and OS of patients with HCC. Since heterogeneity was found among these studies, (I^2^ = 59%, P = 0.04), random-effect model was adopted to calculate the combined HR. Pooled data revealed that elevated PLR was significantly associated with poor OS with a pooled HR of 1.56 (95% CI: 1.13–2.16, P = 0.007; Fig 3), suggesting that elevated PLR was also an indicator of poor survival rate in HCC patients treated with TACE. Every single study was omitted every time to estimate the influence of individual data sets on the pooled HR in Table 2. When removing the study of Sun 2018[48], we observed the heterogeneity between studies was significantly decreased (I^2^ = 19%, P = 0.30), indicating that this study could affect the significance of between-study homogeneity. The sensitive results for the association between preoperative PLR and overall survival were presented in S1 Fig.

**Table 2:** The sensitive analysis results for the association between preoperative PLR and overall survival.

omitting studies	Pooled results of remaining studies			Heterogeneity		
	HR	95%CI	P	I^2^ (%)	P	AEM
Fan 2015	1.49	1.22, 1.81	<0.0001	49	0.12	FEM
He 2019	1.58	1.00, 2.48	0.05	68	0.03	REM
Sun 2018	1.68	1.39, 2.04	<0.0001	19	0.30	FEM
Tian 2016	1.51	1.01, 2.24	0.04	69	0.02	REM
Xue 2015	1.50	0.92, 2.45	0.10	69	0.02	REM

HR, hazard ratio; CI, confidence intervals; FEM, fixed-effects model; AEM, Analytical effect model; REM, random-effects model.

### The prognostic value of preoperative CRP for OS

**Fig 4:**
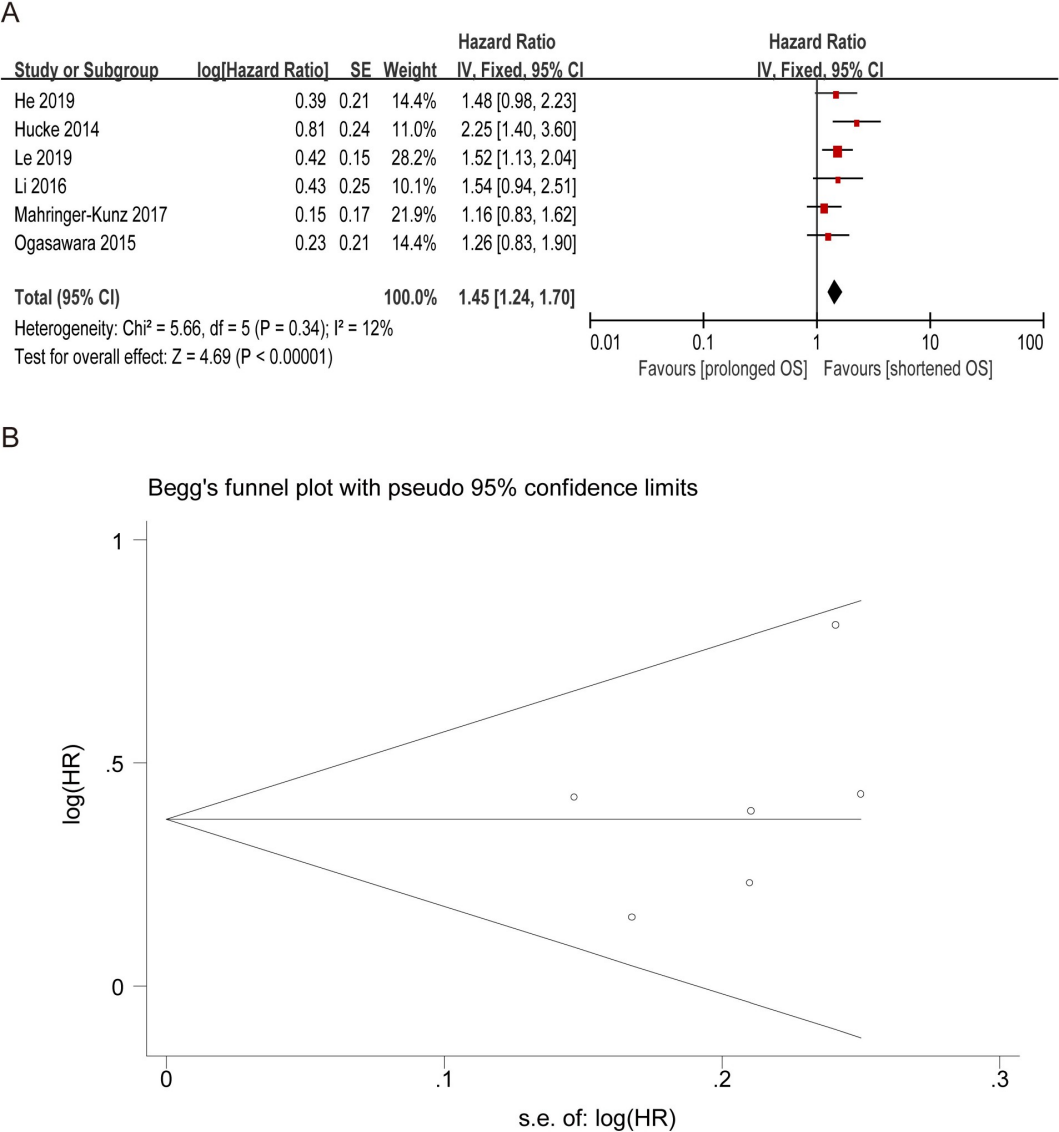
Correlation between CRP and OS of HCC treated by TACE. (A) Forest plot of comparison of the included trials. (B) Funnel plot of comparison of the included trials.

Six studies provided available data for evaluating the prognostic value between CRP and OS of HCC patients undergoing TACE. As no significant heterogeneity between studies was observed (I^2^ = 12%, P = 0.34), fixed-effect model was used to estimate the combined HR of OS. The pooled HR revealed an obvious association between CRP and HCC, with the pooled HR being 1.45 (95% CI: 1.24–1.70, P < 0.00001; Fig 4A). Moreover, the Begg’s funnel plot was symmetric and no publication bias was detected(P = 0.452) (Fig 4B). Therefore, the results indicated that patients with high pretreatment CRP had poor OS.

### Preoperative NLR and clinical features

The associations between NLR and clinical parameters were summarized in Table 3. Six studies provided data about the correlation between elevated NLR and vascular invasion. Pooled results showed that the incidence of elevated preoperative NLR had a significant association with the presence of tumor vascular invasion (OR = 1.49, 95% CI 1.15–1.92, P = 0.002). However, there is no significant correlation between NLR and the other nine clinical features. As no significant heterogeneity between studies was found, fixed-effect models were used except tumor size and serum AFP level.

**Table 3:** The association between incidence of elevated preoperative NLR and clinical features.

Clinical features	Number of studies	Number of patients	OR (95%CI)	P	Effects model	Heterogeneity	
						I^2^ (%)	P_h_
Gender (male vs. female)	9	1655	1.00 (0.76, 1.32)	0.99	FEM	36	0.13
Tumor size (> 5cm vs. < 5cm)	5	971	1.08 (0.60, 1.93)	0.80	REM	55	0.06
BCLC stage (C vs. B)	2	126	0.65 (0.28, 1.50)	0.32	FEM	0	0.52
Vascular invasion (yes vs. no)	6	1333	1.49 (1.15, 1.92)	0.002	FEM	38	0.15
AFP (>400ng/ml vs. <400ng/ml)	6	882	1.15 (0.65, 2.05)	0.62	REM	73	0.002
Child-Pugh class (B vs. A)	7	1356	0.96 (0.74, 1.26)	0.78	FEM	0	0.73
Extrahepatic spread (yes vs. no)	3	376	0.91 (0.55, 1.52)	0.73	FEM	0	0.92
HBV (pos. vs. neg.)	5	627	0.99 (0.64, 1.52)	0.96	FEM	0	0.43
Tumor number (≥ 2 vs. <2)	2	299	1.38 (0.87, 2.19)	0.18	FEM	0	0.88
Tumor number (≥ 3 vs. <3)	2	181	1.17 (0.62, 2.20)	0.63	FEM	33	0.22

OR, odds ratio; CI, confidence intervals; HBV, hepatitis B virus; FEM, fixed-effects model; REM, random-effects model; P_h_: p value of Q test for heterogeneity.

### Preoperative PLR and clinical features

Pooled data of 244 HCC patients showed that high PLR tended to be correlated with higher incidence of tumor size >3 cm (OR = 2.42, 95% CI: 1.31–4.48, P = 0.005). As for the other five clinical features: gender, serum AFP level, Child-Pugh class, vascular invasion, presence of HBV, combined data did not show statistical significance. Since no significant heterogeneity between studies was found, fixed-effect models were used except serum AFP level (Table 4).

**Table 4:** The association between incidence of elevated preoperative PLR and clinical features.

Clinical features	Number of studies	Number of patients	OR (95%CI)	P	Effects mode	Heterogeneity	
						I^2^ (%)	P_h_
Gender (male vs. female)	4	640	0.80 (0.50, 1.26)	0.33	FEM	0	4
Tumor size (> 5cm vs. < 5cm)	2	254	2.42 (1.31, 4.48)	0.005	FEM	0	2
AFP (> 400ng/ml vs. < 400ng/ml)	4	640	1.10 (0.55, 2.19)	0.78	REM	71	4
Child-Pugh class (B vs. A)	3	349	1.21 (0.64, 2.29)	0.55	FEM	12	3
Vascular invasion (yes vs. no)	2	423	0.97 (0.63, 1.48)	0.87	FEM	12	0.29
HBV (pos. vs. neg.)	2	227	0.76 (0.34, 1.69)	0.51	FEM	0	0.86

OR, odds ratio; CI, confidence intervals; HBV, hepatitis B virus; FEM, fixed-effects model; REM, random-effects model; P_h_: P value of Q test for heterogeneity.

## Discussion

At present, inflammation, as a protective response, plays a critical role in the initiation and progression of malignancies, which has aroused widespread interest despite the unclear mechanism. NLR, PLR, and C-reactive protein (CRP) are often used as hematological markers of systemic inflammation to reflect the balance between the host inflammatory response and immune response[39]. The association between inflammation markers (NLR, PLR, CRP) and cancer has already been observed in various types of gastrointestinal malignancies, including esophageal cancer, gastric cancer, colorectal cancer, and pancreatic cancer[38,40–44].

Previously, a meta-analysis demonstrated that elevated preoperative NLR is associated with poor prognosis in HCC patients treated with liver transplantation, and NLR could be used as a marker to predict the survival rate and tumor recurrence rate in HCC patients after liver transplantation[45]. Another meta-analysis indicated that preoperative NLR had significant association with the prognosis of HCC patients underwent curative hepatectomy and may be an effectively prognostic indicator[46]. Moreover, Lin et al. evaluated the prognostic significance of PLR in HCC patients with a total of 2449 patients from 9 studies. The results demonstrated that PLR may be a significant biomarker in the prognosis of HCC in different BCLC stages[7]. CRP is one of the most commonly used indicators for assessing the magnitude of systemic inflammatory response because of its high sensitivity in hospital labor, good specificity, and high reproducibility. Since Hashimoto et al. first demonstrated that the preoperative serum CRP level is an independent and significant factor predictive of a poor prognosis in patients undergoing surgical resection, several investigators have identified an elevated serum CRP level to be an indicator of poor outcomes in HCC patients undergoing transplantation, TACE and radiofrequency ablation[47–51].

This meta-analysis was performed to assess the prognostic value of inflammation markers such as NLR, PLR, and CRP in HCC patients treated with TACE. In the present study, we utilized the available data from 16 included studies with a total of 4023 patients to obtain the pooled results to evaluate the predicted role of NLR in HCC. The pooled outcomes statistically supported the conclusions that elevated NLR predicted poor OS (HR = 1.81, 95% CI: 1.66–1.97, P < 0.00001) in HCC patients treated with TACE. In the subgroup analysis, statistically significance was found respectively in subgroup NLR = 5.0 (HR = 1.74, 95% CI: 1.44–2.11), 2.5≤ NLR <5 (HR = 1.69, 95% CI: 1.50–1.91), and NLR <2.5 (HR = 2.06, 95% CI: 1.77–2.40). The clinical features of HCC, such as tumor multifocality and vascular invasion, are related to the prognosis and survival of HCC[52]. In this case, we performed a pooled analysis to assess the association between elevated NLR and clinical features in HCC. The result indicated that the incidence of high preoperative NLR had significant association with the presence of tumor vascular invasion (OR = 1.49, 95% CI: 1.15–1.92, P = 0.002). In addition, five studies reported evidence about the correlation between elevated PLR and prognosis of HCC patients treated with TACE. Four studies suggested statistical significance, while one studies reported no correlation. Pooled data from all the five studies supported a correlation (HR = 1.56, 95% CI: 1.13–2.16, P = 0.007). Moreover, when we further analyzed the associations between pretreatment PLR and clinicopathologic parameters, we discovered that elevated PLR was linked with tumor size > 3 cm, which was consisted with the results of study Song[53]. Similarly, the pooled outcomes from six included primary studies demonstrated that elevated CRP predicted poor OS in HCC. These results above suggested that elevated preoperative NLR, PLR, and CRP can be used as indicators of poor survival rate in HCC patients treated with TACE.

It is noted that several limitations of this current meta-analysis should be carefully considered. Firstly, considering all the enrolled studies are retrospective, there may be some bias in this meta-analysis, such as information bias, misclassification bias, and selection bias. Secondly, the sample size is so small that only 5 trials were enrolled in the analysis of the correlation between PLR and OS and only 6 trials reported the evidence of the correlation between PLR and OS. Besides, the greatest limitation was the discordance of the cut-off values of the inflammation index used in the included studies. As mentioned earlier, the cut-off value of NLR varies from 1.77 to 5, the cut-off value for PLR ranges from 94.62 to 150, and the cut-off value for CRP ranges from 0.5 to 1 mg/dl. In a way, this difference may account for the heterogeneity between studies. Considering these limitations above, the pooled HR or OR calculated in this study may be just estimation, and more studies that are well-designed, prospective and large-scale are needed to substantiate our results.

In conclusion, we could cautiously come to the conclusion that elevated preoperative NLR, PLR, and CRP are associated with poor prognosis in HCC patients treated with TACE, and they should be used as markers to predict the survival rate and assess the outcomes in HCC patients treated with TACE.

## Supporting information

S1 Fig Forest plot of sensitive results for the association between preoperative PLR and overall survival, which are generalized in Table 2. (TIF)

S1 File The PRISMA flow diagram of this meta-analysis. (DOC)

S2 File The PRISMA checklist of this meta-analysis. (DOC)

S3 File The full search strategy terms for PubMed database. (DOC)
