# Supplementary figures and images for: Prognostic significance of inflammatory indices in hepatocellular carcinoma treated with transarterial chemoembolization: A systematic review and meta-analysis
Source: PLoS One. 2020 Mar 26;15(3):e0230879. doi: 10.1371/journal.pone.0230879 (PMC7098645; doi:10.1371/journal.pone.0230879)

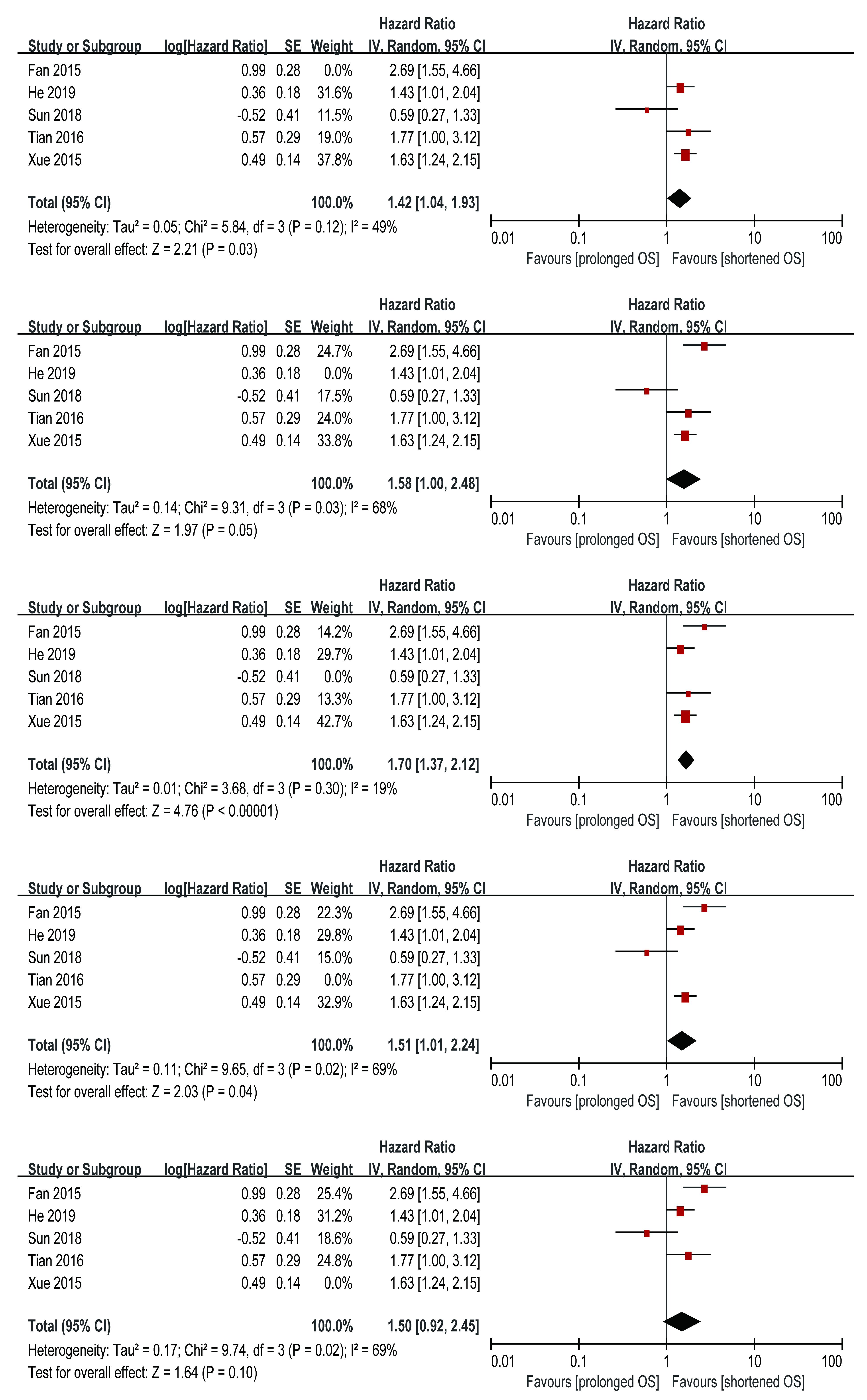

Supplement: S1 Fig — (TIF) [file pone.0230879.s001.tif]
