# Supplementary material for: Prognostic significance of inflammatory indices in hepatocellular carcinoma treated with transarterial chemoembolization: A systematic review and meta-analysis
Source: PLoS One. 2020 Mar 26;15(3):e0230879. doi: 10.1371/journal.pone.0230879 (PMC7098645; doi:10.1371/journal.pone.0230879)
Supplement: S1 File — (DOC) [file pone.0230879.s002.doc]

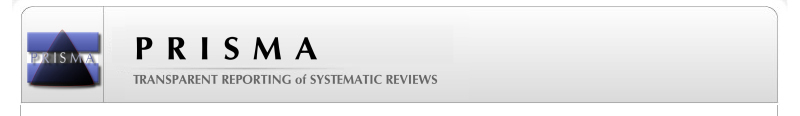
**PRISMA 2009 Flow Diagram**

**Screening**

**Included**

**Eligibility**

**Identification**

Records identified through database searching
(n = 294 )

Additional records identified through other sources
(n = 0 )

Records after duplicates removed
(n = 168 )

Records screened
(n = 168 )

Records excluded
(n = 111 )

Full-text articles assessed for eligibility
(n = 57 )

Full-text articles excluded, with reasons
(n = 35 )

Studies included in qualitative synthesis
(n = 22 )

Studies included in quantitative synthesis (meta-analysis)
(n = 22 )
