# Supplementary material for: Prognostic significance of inflammatory indices in hepatocellular carcinoma treated with transarterial chemoembolization: A systematic review and meta-analysis
Source: PLoS One. 2020 Mar 26;15(3):e0230879. doi: 10.1371/journal.pone.0230879 (PMC7098645; doi:10.1371/journal.pone.0230879)
Supplement: S3 File — (DOC) [file pone.0230879.s004.doc]

Search strategy

PubMed

| No. | Query |
| --- | --- |
| #4 | Search **(#1 AND #2 AND #3)** |
| #3 | Search **(((((((((NLR[Title/Abstract]) OR neutrophil to lymphocyte ratio[Title/Abstract]) OR neutrophil-lymphocyte ratio[Title/Abstract]) OR PLR[Title/Abstract]) OR platelet to lymphocyte ratio[Title/Abstract]) OR platelet-lymphocyte ratio[Title/Abstract]) OR C-reactive protein[Title/Abstract]) OR CRP[Title/Abstract]) OR prognostic nutritional index[Title/Abstract]) OR PNI[Title/Abstract]** |
| #2 | Search **(TACE[Title/Abstract]) OR transarterial chemoembolization[Title/Abstract]** |
| #1 | Search **((((("Carcinoma, Hepatocellular"[Mesh] OR hepatocellular carcinoma[Title/Abstract]) OR HCC[Title/Abstract]) OR liver carcinoma[Title/Abstract]) OR liver cancer[Title/Abstract]) OR Hepatoma[Title/Abstract]) OR Liver Cell Carcinoma[Title/Abstract]** |
